# Purification and Identification of Novel Antioxidant Peptides Isolated from *Geoffroea decorticans* Seeds with Anticoagulant Activity

**DOI:** 10.3390/pharmaceutics13081153

**Published:** 2021-07-27

**Authors:** Juliana Cotabarren, Brenda Ozón, Santiago Claver, Javier Garcia-Pardo, Walter David Obregón

**Affiliations:** 1 Centro de Investigación de Proteínas Vegetales (CIPROVE), Departamento de Ciencias Biológicas, Facultad de Ciencias Exactas, Universidad Nacional de la Plata, Buenos Aires B1900, Argentina; brendaozon@gmail.com (B.O.); claversantiago14@gmail.com (S.C.); 2 Institut de Biotecnologia i Biomedicina and Departament de Bioquimica i Biologia Molecular, Universitat Autònoma de Barcelona, 08193 Barcelona, Spain

**Keywords:** *Geoffroea decorticans*, antioxidant, functional compound, thermostable protein, reactive oxygen species, anticoagulant, bioactive compound

## Abstract

*Geoffroea decorticans* is a xerophilous deciduous tree present in most arid forests of southern South America, which is commonly used in traditional medicine. The seeds of this tree have been previously investigated for their singular chemical composition, but their protein content has been poorly investigated. Herein, we report the isolation, purification, and characterization of a set of thermostable peptides derived from *Geoffroea decorticans* seeds (GdAPs) with strong antioxidant and anticoagulant activities. The most potent antioxidant peptides showed a half maximal inhibitory concentration (IC_50_) of 35.5 ± 0.3 µg/mL determined by 1,1-diphenyl-2-picrylhydrazyl (DPPH). They also caused a dose-dependent prolongation of the aPTT clotting time with an IC_50_ value of ~82 µg/mL. Interestingly, MALDI-TOF/MS analysis showed the presence of three major peptides with low molecular weights of 2257.199 Da, 2717.165 Da, and 5422.002 Da. The derived amino-acid sequence of GdAPs revealed their unique structural features, exhibiting homology with various proteins present in the genome of *Arachis hypogaea*. All in all, our data suggest a direct applicability of GdAPs for pharmaceutical purposes.

## 1. Introduction

Over many centuries, plants have historically made a major contribution to pharmacology. In fact, an important number of currently available drugs are derived directly or indirectly from natural products, especially as antibacterial or antitumor agents [1,2]. However, there is growing evidence that modern pharmacology has only explored and validated a small part of this large array of molecular entities. The best-characterized examples of bioactive molecules derived from plants include peptides, proteins, sugars, nucleosides, or products of the secondary metabolism of the plant. Among them, plant-derived peptides have shown a variety of interesting biological activities such as hypertensive, hypoglycemic, antioxidant, and/or anticoagulant properties. This broad bioactivity spectrum of these peptide molecules makes them attractive for the treatment or prevention of various diseases [3,4,5].

In recent years, substantial research efforts were dedicated toward the discovery of novel drugs to treat cardiovascular diseases (CVDs). CVDs are a group of disorders of the heart and blood vessels that represent one of the major causes of mortality worldwide. In most cases, the drugs used to treat CVDs are anticoagulant molecules that inhibit or delay coagulation by acting on one or more coagulation factors. Coagulation and fibrinolysis are interrelated enzymatic processes that, together with mechanisms involving the vascular wall and platelets, keep the blood flowing within the blood vessels. An inadequate activation of coagulation or the uncontrolled activation of fibrinolysis can generate hemorrhagic disorders, such as in Von Willebrand’s disease, hemophilia, or hemolytic anemia [6]. It is known that a cascade of enzyme activations occurs in the coagulation process, which are mainly regulated by inhibitors of a protein nature [7]. Current pharmacological agents used to prevent or treat thrombotic disorders are mostly synthetic antiplatelet, anticoagulants, and fibrinolytic agents [8], which remain under continuous review due to their adverse effects and bleeding risks [9,10]. Thus, the identification of novel anticoagulant compounds from natural sources has gained interest because of their direct application in the treatment of CVDs and other associated diseases such as viral infections [11], cancer [12], malaria [13], hypertension [14,15], and/or blood disorders [16]. On the other hand, it has been previously demonstrated that reactive oxygen species (ROS) play a vital role in the development and progression of CVDs [17,18,19,20,21]. In fact, generation of ROS and free radicals have deleterious effects on many molecules in the human body, such as DNA, fatty acids, and proteins [22]. Typically, proteins and peptides can inhibit oxidative reactions, in part due to their ability in neutralizing the effects of transition metals, leading to the formation of less reactive insoluble metal complexes [23]. Recent studies have shown that crude proteins from some of the seeds of plant species such as pea [24], chickpea [25], and peanut kernels [26] display strong antioxidant activity associated with a low toxicity profiles [27].

A growing number of bioactive peptides have been identified from various medicinal plant sources [28,29,30,31]. *Geoffroea decorticans* (Gill. ex Hook. et Arn.) Burkart (Fabaceae) is a xerophilous deciduous tree mostly found in arid areas of South America used in traditional medicine. This plant is also known commonly as chañar. Chañar fruits have been consumed fresh or cooked by human communities since ancient times [32,33]. The fruits of *G. decorticans* are brown spherical drupes with sweet pulp and palatable taste. The edible part is composed of the fleshy mesocarp that, air-dried, contains 11.10% sugars and 4.49% resins with 8.75% protein, 15.20% reducing sugar, and 52.89% starch and other carbohydrates. Chañar seeds are also edible (both fresh or roasted) with about 22% protein and a high oil content of about 47%, where the oleic–linoleic ratio stands out (53.7%:30.7%) [34]. In addition, some studies have reported antifungal properties in the organic extracts of the chañar stem bark, especially associated with inflammatory processes [35,36,37,38]. Despite all these previous studies, the protein composition and activity of the bioactive compounds present in this plant have been poorly characterized.

Herein, we report the isolation and characterization of a set of thermostable bioactive peptides obtained from *G. decorticans* seeds. These native peptides showed a potent antioxidant activity in vitro. Furthermore, we demonstrate the suitability of these bioactive peptides as anticoagulant molecules targeting the intrinsic pathway of coagulation. Taken together, the current results provide useful information for the production of novel plant-derived anticoagulant molecules with additional functionalities.

## 2. Materials and Methods

### 2.1. Materials

#### 2.1.1. Chemicals

Coomassie Blue G-250, sodium chloride, tris(hydroxymethyl) aminomethane, sodium dodecyl sulfate (SDS), *N*,*N*,*N*′,*N*′-tetramethylethylenediamine (TEMED), β-mercaptoethanol (βME), 1,1-diphenyl-2-picrylhydrazyl (DPPH), bovine serum albumin (BSA) and 2,2′-azino-bis(3-ethylbenzothiazoline-6-sulfonic acid) diammonium salt (ABTS) were purchased from Sigma-Aldrich (Saint Louis, MO, USA). Glyoxyl-agarose was purchased from FlukaTM (Charlotte, NC, USA).

#### 2.1.2. Sample Collection

The ripe fruits of *Geoffroea decorticans* were hand-collected from 20 trees located in the department of Juan Martín de Pueyrredón (33°16′02.9′′ S 66°12′50.2′′ W), Juana Koslay, San Luis province, Argentina. The samples were collected in January 2018 and identified as *Geoffroea decorticans* (Gill., ex Hook & Arn, Burkart, family Fabacea) by specialists of the Facultad de Química, Bioquímica y Farmacia, Universidad Nacional de San Luis, Argentina. A voucher specimen was deposited at the Herbarium of the Universidad Nacional de San Luis (UNSL Del Vitto N° 553). *G. decorticans* fruits were flash-frozen and stored at −20 °C until use.

### 2.2. Crude Extract Preparation and Determination of the Protein Content

Chañar seeds were obtained from *G. decorticans* (chañar) fruits after the woody endocarp was hand-broken and removed. To prepare the crude extract, 10 g of seeds were washed with distilled water. The sample was ground using a blender in 100 mL of 0.1 M Tris–HCl buffer, pH 7.5. This process was performed on an ice bath to reduce protein denaturation. After incubation for 120 min at 4 °C, the mixture was filtered through a fine screen cloth and centrifuged for 30 min at 7000× *g* at 4 °C. Afterward, the supernatant (hereafter GdCE) was collected and immediately flash-frozen at −20 °C.

The soluble protein was determined using Bradford’s assay [39]. Briefly, to perform the reactions, 10 µL of each standard solution or test sample was mixed with 200 µL of the Bradford reagent. After 10 min incubation at room temperature, the absorbance at 595 nm was recorded using a Tecan Infinite M200 PRO spectrophotometer (Männedorf, Switzerland). Bovine serum albumin (BSA) was used as a protein concentration standard. This method is particularly suitable for the determination of protein content of vegetable extracts, which usually have phenolic compounds that interfere with the traditional Lowry method. All the measurements of the soluble protein content were carried out in triplicate.

### 2.3. Purification of Antioxidant Peptides

#### 2.3.1. Initial Purification by Heat Treatment

The crude extract (GdCE) was subjected to a water bath at 90 °C for 30 min. After cooling the sample, precipitated proteins were removed by centrifugation at 4 °C for 30 min at 7000× *g*. After centrifugation, the soluble protein content and the antioxidant activity of the nontreated crude extract and the heat-treated sample (GdHT) were determined. SDS-PAGE analysis was used to determine the protein profile during the different purification steps. Gels were silver-stained following the Chevallet, Luche, and Rabilloud [40] protocol. Densitograms for each lane were produced using the GelAnalyzer 19.1 program. The obtained peaks were compared according to computed Rf (Retardation factor) values and band intensity, which is proportional to the protein concentration on each peak.

#### 2.3.2. Size-Exclusion Chromatography

A 1 mL aliquot of the heat-treated sample (1767 µg/mL) was loaded onto a Sephacryl-S100 HR column (1.5 × 40 cm) connected to an Äkta-Purifier (GE Healthcare) previously equilibrated with milliQ water. Elution was carried out using an isocratic gradient and a flow rate of 0.8 mL/min. During elution, the absorbance was continuously monitored at 280 and 215 nm. The antioxidant activity was determined for each fraction, and the fraction with the higher antioxidant activity was selected for further analysis.

### 2.4. MALDI-TOF Mass Spectrometric Analysis

To perform mass spectrometry (MS) analysis, an aliquot of the sample from the exclusion chromatography was desalted by dialysis in 0.022 µm filters. The dialyzed sample was mixed with an equal volume of 3,5-dimethoxy-4-hydroxycinnamic acid (sinapinic acid) matrix solution. Then, the matrix–sample mixture was spotted onto an MTP 384 target plate (polished steel TF, Bruker Daltonics, Billerica, MA, USA). After the spots were evaporated to dryness at room temperature, the mass spectra were acquired on a Bruker Daltonics Ultraflex MALDI-TOF mass spectrometer (Bruker Daltonics) operating in linear positive and reflector mode.

The PMF analysis was performed for identification of the antioxidant peptides of Chañar. First, an in situ tryptic digestion of the Tris–tricine SDS-PAGE electrophoresis lane containing the protein bands was performed by following a standard PMF protocol [41]. In brief, the tryptic peptides were dissolved in 10 µL of 0.1% CF3CO2H (*v*/*v*) and analyzed by MALDI-TOF in the presence of an HCCA matrix. Mass spectra of the tryptic peptides were acquired using a Bruker Daltonics Ultraflex MALDI-TOF mass spectrometer (Bruker Daltonics) in a reflectron positive mode operating at 25 kV acceleration voltage. In both cases, a standard peptide calibration mixture from Bruker Daltonics used as reference. Four precursor ions MH^+^ (1267.672, 1682.694, 1925.806, and 2778.353) were subjected to TOF–TOF fragmentation. The resultant fragmentation spectra for these precursor MH^+^ ions were compared with all the fragmentation patterns for all the sequences present in the MASCOT database, considering the carbamidomethylation of all Cys (www.matrixscience.com, access on 1 April 2020). All the MS experiments were performed at the Proteomics Core Facility CEQUIBIEM, at the University of Buenos Aires/CONICET (National Research Council).

### 2.5. Antioxidant Activity Evaluation

#### 2.5.1. DPPH Radical-Scavenging Activity

The Brand-Williams method was used to evaluate the DPPH scavenging activity against free radicals [42]. The method was adapted to a 96-well flat-bottom plate. In essence, 50 μL of each sample were mixed with 50 μL of a 0.2 mmol/L DPPH radical solution and 150 μL of methanol. After incubation for 20 min at 37 °C in dark conditions, the absorbance of each well was measured at 517 nm using a microplate spectrophotometer. As a control condition, the maximum DPPH absorbance was determined by replacing the sample volume with the same volume of water. For IC_50_ determination, six different concentrations for each sample were analyzed. Measurements were carried out in triplicate. The IC_50_ values were calculated using GraphPad software. DPPH radical-scavenging activity was calculated using the following equation:

$$\mathrm{DPPH}\;\mathrm{radical}\;\mathrm{scavenging}\;\mathrm{activity}\;\left(\%\right)=100\times \frac{A1-A2}{A1},$$

where A1 is the absorbance of the control without sample and A2 is the absorbance in presence of sample and DPPH.

#### 2.5.2. ABTS Radical-Scavenging Activity

The ABTS^+^ radical-scavenging activity was determined using the method described by Pukalskas et al. [43], with slight modifications. The antioxidant activity in the samples was analyzed by adding 10 μL of sample to 190 μL of diluted ABTS^+^ solution. After 10 min incubation at room temperature in the darkness, absorbance at 734 nm was measured using a microplate spectrophotometer. Measurements were carried out in triplicate. For IC_50_ determination, six different concentrations of each sample were analyzed. All the assays were carried out in triplicate. The IC_50_ values were calculated using GraphPad software. The ABTS^+^ radical-scavenging activity was calculated using the following equation:

$$\mathrm{ABTS}\;\mathrm{radical}\;\mathrm{scavenging}\;\mathrm{activity}\;\left(\%\right)=100-100\times \frac{A1-A2}{A0},$$

where A0 is the absorbance of the control without sample and A1 is the absorbance in the presence of the sample and ABTS^+^. A2 corresponds to the value of absorbance of sample blank without ABTS^+^.

### 2.6. Anticoagulant Activity

The anticoagulant activity of GdAPs was evaluated as previously described by our group [44]. In essence, we measured the prothrombin time (PT) and the time of activated partial thromboplastin (aPTT) using a Coatron M1 coagulometer (TECO, Germany). Before performing the experiments, a pool of blood plasmas obtained from five different samples of healthy individuals was prepared, aliquoted, and stored at 37 °C with 3.8% sodium citrate (sample–anticoagulant ratio of 9:1). This reference sample was used in both determinations (hereafter PBP) to evaluate the PT and aPTT time. For the PT test, the commercial Soluplastin reactive was used (Wiener Lab.) To perform reactions, equal parts of the sample PBP and GdAPs (0–215 μg/mL) were incubated for 2 min at 37 °C. Afterward, 50 μL of Soluplastin were added to 25 μL of the mixture and the sample was evaluated in order to determine the coagulation time. For the aPTT test, 25 μL of aPTT (Wiener Lab.) was added to an equal volume of PBP–GdAPs mixture (previously incubated for 2 min at 37 °C). After incubation for 2 min at 37 °C, 25 μL of 50 mM CaCl_2_ was added to the samples. All the experiments were performed at least in triplicate.

### 2.7. Statistical Analysis

Statistical analyses (ANOVA) were performed using GraphPad Prism v. 01 software (http://www.graphpad.com/scientific-software/prism/, access on 10 June 2021). A Tukey’s post hoc test (*p* < 0.05) was used to identify significant differences between the means of the different groups analyzed.

## 3. Results and Discussion

### 3.1. Isolation and Purification of the Antioxidant Peptides

**Figure 1:**
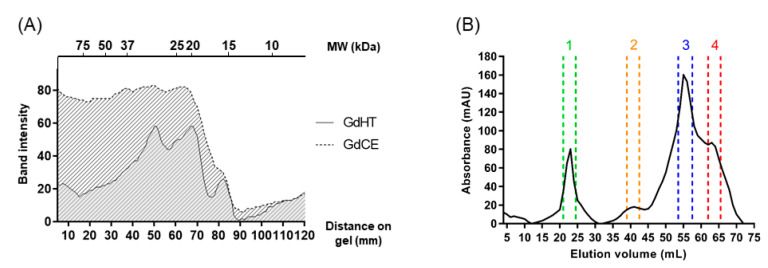
Isolation and purification of antioxidant peptides from *Geoffroea decorticans* seeds. (**A**) Densitogram of SDS-tricine-PAGE of the crude extract of *Geoffroea decorticans* (GdCE, dotted line) and the heat-treated sample (GdHT, solid line). (**B**) Size-exclusion chromatography elution profile of the *Geoffroea decorticans* heat-treated sample. Fractions 1–4 selected for posterior analysis are shown between dotted lines.

A crude extract (GdCE), with a protein concentration of 1069 µg/mL, was obtained from chañar seeds. As a second step of purification, the crude extract was subjected to a heat treatment (hereafter referred to as GdHT), which had a lower protein concentration than the crude extract (489 µg/mL). Figure 1A shows the purification and densitogram analysis of the electrophoretic profile of the proteins present in the crude extract and the heat-treated sample. In essence, this analysis revealed that the thermal treatment produces a removal of heat-labile proteins, with molecular masses over 20 kDa, according to the concentration values obtained by the Bradford assay. Thus, the incubation of the crude extract at high temperatures generated a clarified product that was more suitable for the next purification steps, where there was a higher proportion of low-molecular-weight proteins than in the crude extract. It is worth mentioning that heat treatment does not affect the stability of low-molecular-weight proteins, as previously reported for plant protease inhibitors that preserve their biological activity after thermal treatments [4,5].

**Figure 2:**
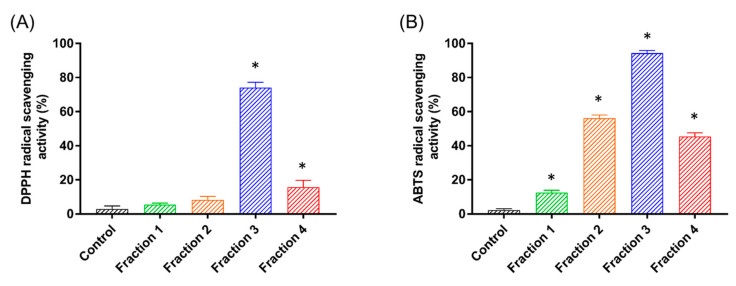
Antioxidant activity of the major peptides fractions isolated *Geoffroea decorticans* seeds. (**A**) DPPH radical-scavenging activity. (**B**) ABTS radical-scavenging activity. The vertical bars correspond to the standard deviation. Fractions 1–4 were used at the same concentration. * *p* < 0.05, compared to DPPH/ABTS control reactions (control) without sample.

Next, we aimed to evaluate the antioxidant capacity of the crude extract and the heat-treated sample in order to monitor the presence of peptides with antioxidant activity in both fractions. This initial analysis showed that the IC_50_ values for GdHT (IC_50_ by DPPH = 63.2 ± 0.6 µg/mL; IC_50_ by ABTS = 141.3 ± 3.1 µg/mL) were lower than those obtained for GdCE (IC_50_ by DPPH = 82.5 ± 1.3 µg/mL; IC_50_ by ABTS = 175.7 ± 4.5 µg/mL). Subsequently, 1 mL of GdHT in 1767 µg/mL concentration was loaded into a size-exclusion column, and the eluted fractions were monitored by absorbance at 280 nm. As shown in Figure 1B, the initial sample was separated into four different fractions. A detailed analysis of this profile revealed a predominance of a fraction that eluted with a retention volume close to 60 mL, corresponding to low-molecular-weight proteins. Then, we determined the antioxidant activity on 20 µg/mL of the four fractions obtained by size-exclusion chromatography (see Figure 2). In this way, it was possible to identify the presence of peptides with a strong antioxidant activity in fractions 2, 3, and 4. However, the major activity was found in fraction 3 (see Figure 2). Typically, low-molecular-weight peptides are related to a greater antioxidant capacity [45,46,47,48]. However, in this case, fraction 3 had higher antioxidant activity compared to fraction 4. This difference could be attributed to the singular peptide composition of each fraction. Furthermore, it was possible to appreciate differences between the antioxidant activities obtained by the ABTS method with respect to that observed for DPPH. These differences, which were also observed in the crude and the heat-treated extracts, can be explained by the difference in the diffusivity of the radicals in the reaction medium; while ABTS radicals are soluble in both organic solvents and aqueous solutions, DPPH radicals are only soluble in organic medium [49]. Accordingly, fraction 3 (from now on GdAPs) was selected for the following biochemical analyses.

### 3.2. Identification and Antioxidant Properties of GdAPs

**Figure 3:**
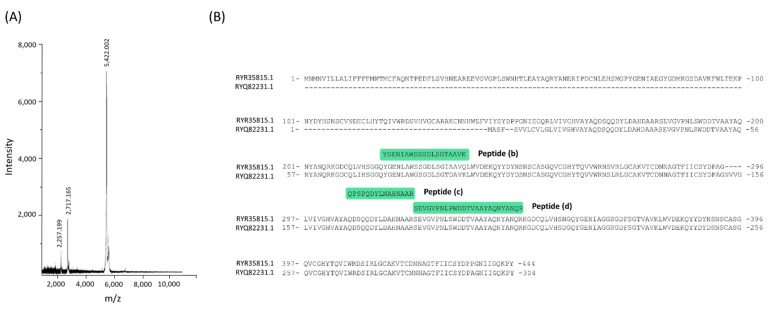
Peptide identification by MALDI-TOF mass spectrometric analysis. (**A**) MALDI-TOF-MS spectrum of GdAPs recorded in a linear positive mode (mass range from 0.9 to 10 kDa). (**B**) Sequence alignment of the tryptic peptides identified by MALDI-TOF MS/MS with the proteins RYR35815.1 and RYQ82231.1 encoded in the *Arachis hypogaea* genome.

The protein fraction with the highest antioxidant activity was subjected to MALDI-TOF mass spectrometry analysis (see Section 2 for details). As shown in Figure 3, the MS spectra revealed the presence of three predominant low-molecular-weight peaks with apparent molecular masses of 2257.199 Da, 2717.165 Da, and 5422.002 Da (see Figure 3). This result indicates that fraction 3 is enriched in a set of peptides in the range of 2–8 kDa, with a predominance of three major peptide species. In order to identify the antioxidant peptides present in the sample, the bands corresponding to the three major peptides observed on the SDS-PAGE were submitted to tryptic digestion. The resulting peptides mixture obtained by tryptic digestion was evaluated by MALDI-TOF–TOF MS/MS in order to determine their amino-acid sequences. From these analyses it was possible to solve the sequence of four different tryptic peptides corresponding to (a) a 1267.672 Da peptide: EHIMPLGQNGR, (b) a peptide of 1682.694 Da: QPSPQDYLNAHNAAR, (c) a 1925.806 Da peptide: YGENIAWSSGDLSGTAAVK, and (d) a 2778.353 Da peptide: SEVGVPNLPWDDTVAAYAQNYANQR. When the amino-acid sequence of peptide (a) was compared to NCBI’s nonredundant database, no similarity was found with any of the peptide sequences deposited in the database. For sequences (b), (c), and (d), the database identified them with several hypothetical proteins from other plants of the Fabaceae family (see Table S1). The sequence alignment between the sequences deposited in the database and the peptides (b), (c), and (d), shows that these three peptides correspond to various regions found in hypothetical proteins obtained from the *Arachis hypogaea* genome (Figure 3). These proteins arise from the sequencing of the complete genome of the plant of origin and represent proteins predicted on the basis of the genomic sequence, without having been isolated, purified, and/or characterized so far. Therefore, this result is in agreement with the presence of newly discovered bioactive molecules in *G. decorticans* that have not been previously characterized.

**Figure 4:**
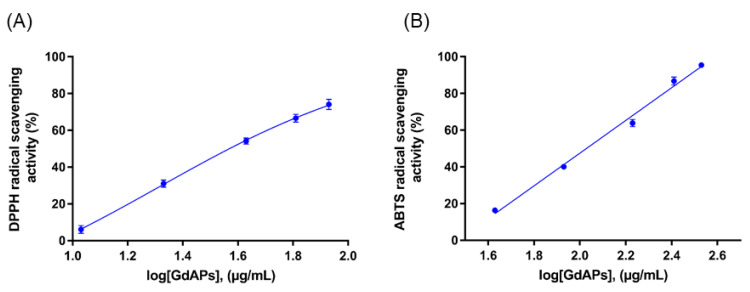
Antioxidant activity of *Geoffroea decorticans* antioxidant peptides. (**A**) DPPH radical-scavenging activity. (**B**) ABTS radical-scavenging activity. The vertical bars correspond to the standard deviation of three independent measurements (*n* = 3).

Previous studies have shown that the amino-acid composition of the peptides has an important effect on their antioxidant activity [50]. It is common to find a high representation of amino acids such as His, Trp, Phe, Pro, Gly, lys, Ile, and Val in antioxidant peptides. Their residues are endorsed with intrinsic radical-scavenging activity in oxidative reactions, especially for those enzyme-catalyzed reactions. In the case of Trp, the presence of an imidazole ring is an important chemical feature [51]. Another study also suggested that the antioxidant capacity of peptides could be notably enhanced by the overrepresentation of three aromatic amino acids such as Trp, Tyr, and Pre [52]. In addition, the indole and pyrrolidine rings present in Trp and Pro, respectively, could also serve as good hydroxyl radical scavengers. Other amino acids, i.e., Gly, Lys, Ile, and Val, may be responsible for creating a favorable hydrophobic environment for peptide molecules. As observed from the partial amino-acid sequence obtained for GdAPs, the four identified peptides exhibited hydrophobic amino acids in their sequence, in accordance with the amino-acid composition that is characteristic for antioxidant peptides [50,51,52]. Lastly, considering the relevance for the biomedical field on the description of novel natural antioxidant molecules, a detailed characterization of the antioxidant capacity of GdAPs was carried out by determining the concentration IC_50_ values of these peptides. Again, we used both ABTS and DPPH methods to determine the antioxidant activity of the peptides at five different concentrations (see Figure 4). The resultant IC_50_ values were 95.1 ± 0.6 µg/mL and 35.5 ± 0.3 µg/mL for the ABTS and DPPH methods, respectively (see Figure 4). These values are in the range of the IC_50_ values of other antioxidant peptides derived from natural origin [28,29,30,31]. Furthermore, the results of the IC_50_ obtained for GdAPs with respect to the values for GdHT show that they decreased by half for both methods, which is consistent with the purification obtained by means of molecular exclusion chromatography (see Table 1).

The purification methodologies used here yielded 14.1% and a 2.3 and 1.9 purification fold when analyzed by DPPH and ABTS, respectively, similar to the purification fold observed in previous studies [53]. Therefore, the production of antioxidant peptides from chañar seeds is potentially feasible to be scaled at an industrial level, since it does not require large investment or very extensive technological processes. Most of the antioxidant peptides reported to date correspond to peptides generated by enzymatic hydrolysis [45,48]. However, purifying native antioxidant peptides may have several advantages in comparison with the obtention of peptides produced by enzymatic hydrolysis. These advantages include the presence of low levels of allergens or the absence of bitter taste in the final product(s) [54,55]. The major antioxidant peptides present in *G. decorticans* are not only within the lower range of molecular weight obtained for other peptides, but also have a greater antioxidant capacity than other native peptides/proteins reported to date, such as GSQ [28], G4b [53], QCZDE, QCZCM [56], TCP-III [31], and APC [29] (Table 2).

### 3.3. GdAPs Display Strong Anticoagulant Activiy against the Intrinsic Pathway

The diseases associated with the blood circulation system are the main causes of mortality in many countries all over the world [59]. In this context, anticoagulants such as heparin, coumarine, or warfarin have been widely used for the treatment of arterial and venous thrombotic disorders, as well as in preventive medicine. However, these common anticoagulant drugs present some limitations such as their inefficacy in antithrombin-deficient patients, bleeding complications, and/or the potential development of drug-induced thrombocytopenia and immunosuppression. The presence of these complications has prompted scientists to exploit natural products as a solid alternative to currently available anticoagulants [60].

**Figure 5:**
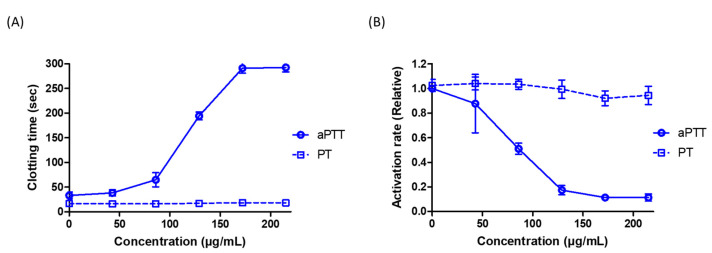
Anticoagulant activity of GdAPs on activated partial thromboplastin (aPTT) and prothrombin (PT). (**A**,**B**) Effect of GdAPs concentration on the clotting time and (**B**) activation rate of aPTT and PT. The vertical bars correspond to the standard deviation of three independent measurements (*n* = 3).

Herein, we assessed the activity of the purified GdAPs in an activated partial thromboplastin time (aPTT) assay. Using this approach, we evaluated the activation of the intrinsic and common pathways of the coagulation cascade as the time taken for fibrin clot formation. As shown in Figure 5A, the purified GdAPs caused a prolongation of the clotting time in a dose-dependent manner. An important key parameter that is drawn from this in vitro assay is the dose of the anticoagulant that provides two-fold prolongation of clotting time compared with the untreated samples. This allows measuring the effect of our peptides against factors VIII, IX, XI, and XII and Prekallikrein of the coagulation cascade. GdAPs showed a twofold prolongation time at concentrations in the range 86–215 µg/mL, with an IC_50_ value of ~82 µg/mL (see Figure 5B). Interestingly, the prothrombin time (PT) was not significantly increased in the presence of GdAPs in the range of concentrations assayed (IC_50_ > 215 µg/mL). As a comparison, heparin, a drug commonly used in clinical applications, in a concentration of 2 mg·mL^−1^, produces a delay in coagulation time for the aPTT route from 18 to 300 s [61], while GdAPs produces the same effect at much lower concentrations of 172 µg·mL^−1^. This behavior was previously reported for other peptides from plant origin such as the trypsin inhibitors from *Leucaena leucocephala* [62], *Enterolobium contortisiliquum* [63], and *Maclura pomifera* [64].

In summary, the presented results show that GdAPs have significant beneficial effects as an anticoagulant drug. In addition, our results demonstrate that these peptides target the intrinsic pathway, leading to a retardation of the clotting time. Taken together, our results encourage future studies on the mechanism of action of GdAPs and the development of in vivo assays that could be promising for a potential exploitation of these peptides as naturally occurring antioxidant and antithrombotic agents.

## 4. Conclusions

Plants represent a rich source of bioactive compounds suitable for pharmaceutical applications. Although *G. decorticans* is a native plant endemic of South America that is recognized for its medicinal properties, only few reports of bioactive molecules isolated from this plant source are available so far [44]. In this study, we successfully isolated a set of native peptides present in *G. decorticans* seeds. To achieve this, the seed extracts were subjected to simple purification steps, such as heat treatment and molecular exclusion chromatography. Other previous reports used enzymatic hydrolysis with proteases as biocatalysts, which requires multiple time-consuming steps and additional reagents for the production of the bioactive peptides. Interestingly enough, these peptides have remarkable thermal stability, which is an added benefit of their conservation without the need for refrigeration. This would allow their use to be adapted to many geographical regions of the world that have a shortage of technologies and adequate infrastructure for storage. Lastly, we demonstrated that the peptides present in *G. decorticans* have a strong antioxidant capacity, comparable to other previously isolated molecules (see Table 2). More interestingly, we revealed that such molecules have a strong anticoagulant effect against the intrinsic coagulation pathway. All in all, our findings position *G. decorticans* as an attractive source of bioactive molecules and suggest a possible biomedical application of GdAPs isolated from the seeds of this plant.

## Figures and Tables

**Table 1:** Purification steps and antioxidant activity of GdAPs from *Geoffroea decorticans* seeds.

Purification Step	Soluble Protein (mg)	Yield	Inhibition of DPPH IC_50_ (µg/mL)	Purification Fold ^(a)^	Inhibition of ABTS IC_50_ (µg/mL)	Purification Fold ^(b)^
Crude extract	21.4 ± 0.4	100	82.5 ± 1.2	1	175.7 ± 3.1	1
90 °C heat treatment	17.7 ± 0.5	82.6 ± 0.7	63.2 ± 0.8	1.3 ± 0.1	141.3 ± 2.7	1.2 ± 0.1
Size-exclusion chromatography	3.1 ± 0.2	14.1 ± 0.3	35.5 ± 0.5	2.3 ± 0.1	95.1 ± 0.9	1.9 ± 0.1

^(a)^ Purification fold determined for DPPH inhibition; ^(b)^ purification fold determined for ABTS inhibition.

**Table 2:** Native antioxidant peptides/proteins isolated from plant sources in the last decade.

AntioxidantPeptide/Protein	Plant Source	MW (Da)	IC_50_ by DPPH (µg/mL)	IC_50_ by ABTS (µg/mL)	References
GSQ	*Allium tuberosum* Rottler	290.10	610	40	[28]
G4b	*Ginkgo biloba* seeds	29,247	100.7	n/d	[53]
QCZDE	*Apium graveolens* seeds	6500	n/d	158.7	[56]
QCZCM	*Apium graveolens* seeds	11,390	n/d	193.2	[56]
Gg-AOPI	*Gnetum gnemon* seeds	30,000	21	10	[30]
Gg-AOPII	*Gnetum gnemon* seeds	12,000	27	12	[30]
Antioxidant protein from Curcuma comosa	*Curcuma comosa* Roxb. Rhizomes	18,000	n/d	n/d	[57]
PNP	*Phyllanthus nuri* leaves	35,000	n/d	n/d	[58]
TCP-III	*Terminalia chebula* fruit	16,267	291	n/d	[31]
APC	*Murraya koenigii* leaves	35,000	70	n/d	[29]
GdAPs	*Geoffrea decorticans*	2000–8000	35.5	95.1	-

Abbreviations: n/d, not determined.

## Data Availability

Not applicable.

## Supplementary Materials

The following are available online at https://www.mdpi.com/article/10.3390/pharmaceutics13081153/s1, Table S1: Protein sequences obtained from the NCBI database after performing an sequence similarity search for the peptides (b), (c) and (d).
