# Supplementary material for: Purification and Identification of Novel Antioxidant Peptides Isolated from Geoffroea decorticans Seeds with Anticoagulant Activity
Source: Pharmaceutics. 2021 Jul 27;13(8):1153. doi: 10.3390/pharmaceutics13081153 (PMC8399481; doi:10.3390/pharmaceutics13081153)
Supplement: Supplementary file 1 [file pharmaceutics-13-01153-s001.zip › pharmaceutics-1250247-final.pdf]

pharmaceutics

MDPI

Supplementary Information: Purification and Identification of Novel Antioxidant Peptides Isolated from *Geoffroea decorticans* Seeds with Anticoagulant Activity

Juliana Cotabarren, Brenda Ozón, Santiago Claver, Javier Garcia-Pardo and Walter David Obregón

Citation: Cotabarren, J.; Ozón, B.; Claver, S.; Garcia-Pardo, J.; Obregón, W.D. Purification and Identification of Novel Antioxidant Peptides Isolated from *Geoffroea decorticans* Seeds with Anticoagulant Activity. *Pharmaceutics* **2021**, *13*, 1153. https://doi.org/10.3390/pharmaceutics13081153

Academic Editor: Maria Rosaria Lauro

Received: 14 June 2021
Accepted: 22 July 2021
Published: 27 July 2021

Publisher's Note: MDPI stays neutral with regard to jurisdictional claims in published maps and institutional affiliations.

Copyright: © 2021 by the authors. Submitted for possible open access publication under the terms and conditions of the Creative Commons Attribution (CC BY) license (http://creativecommons.org/licenses/by/4.0/).

Table S1. Protein sequences obtained from the NCBI database after performing an sequence similarity search for the peptides (b), (c) and (d).

Organism	Query Cover	E Value	Per. Ident.
Arachis duranensis	100%	$1 \times e^{-6}$	93.33%
Arachis hypogaea	100%	$1 \times e^{-6}$	93.33%
Vigna unguiculata	100%	$2 \times e^{-5}$	86.67%
Vigna unguiculata	100%	$2 \times e^{-5}$	86.67%
Abrus precatorius	100%	$2 \times e^{-5}$	86.67%
Arachis hypogaea	100%	$2 \times e^{-9}$	94.44%
Arachis duranensis	94%	$2 \times e^{-9}$	94.44%
Arachis hypogaea	94%	$2 \times e^{-9}$	94.44%
Prosopis alba	100%	$1 \times e^{-8}$	84.21%
Arachis duranensis	100%	$2 \times e^{-6}$	84.21%
Arachis hypogaea	100%	$5 \times e^{-18}$	96%
Arachis hypogaea	100%	$5 \times e^{-18}$	96%
Arachis duranensis	100%	$5 \times e^{-18}$	96%
Arachis ipaensis	100%	$5 \times e^{-18}$	96%
Arachis ipaensis	100%	$5 \times e^{-18}$	96%
